# Determinants of RSV epidemiology following suppression through pandemic contact restrictions

**DOI:** 10.1016/j.epidem.2022.100614

**Published:** 2022-07-21

**Authors:** Mihaly Koltai, Fabienne Krauer, David Hodgson, Edwin van Leeuwen, Marina Treskova-Schwarzbach, Mark Jit, Stefan Flasche

**Affiliations:** a Department for Infectious Disease Epidemiology, London School of Hygiene & Tropical Medicine, London, UK; b Centre for Mathematical Modelling of Infectious Diseases, London School of Hygiene & Tropical Medicine, London, UK; c Statistics, Modelling and Economics Department, UK Health Security Agency, London, UK; d Robert Koch-Institute, Berlin, Germany

**Keywords:** RSV, Epidemiology, Non-pharmaceutical interventions, Disease Dynamics, Transmission modelling

## Abstract

**Introduction:**

COVID-19 related non-pharmaceutical interventions (NPIs) led to a suppression of RSV circulation in winter 2020/21 in the UK and an off-season resurgence in Summer 2021. We explore how the parameters of RSV epidemiology shape the size and dynamics of post-suppression resurgence and what we can learn about them from the resurgence patterns observed so far.

**Methods:**

We developed an age-structured dynamic transmission model of RSV and sampled the parameters governing RSV seasonality, infection susceptibility and post-infection immunity, retaining simulations fitting the UK’s pre-pandemic epidemiology by a set of global criteria consistent with likelihood calculations. From Spring 2020 to Summer 2021 we assumed a reduced contact frequency, returning to pre-pandemic levels from Spring 2021. We simulated transmission forwards until 2023 and evaluated the impact of the sampled parameters on the projected trajectories of RSV hospitalisations and compared these to the observed resurgence.

**Results:**

Simulations replicated an out-of-season resurgence of RSV in 2021. If unmitigated, paediatric RSV hospitalisation incidence in the 2021/22 season was projected to increase by 30–60% compared to pre-pandemic levels. The increase was larger if infection risk was primarily determined by immunity acquired from previous exposure rather than age-dependent factors, exceeding 90 % and 130 % in 1–2 and 2–5 year old children, respectively. Analysing the simulations replicating the observed early outbreak in 2021 in addition to pre-pandemic RSV data, we found they were characterised by weaker seasonal forcing, stronger age-dependence of infection susceptibility and higher baseline transmissibility.

**Conclusion:**

COVID-19 mitigation measures in the UK stopped RSV circulation in the 2020/21 season and generated immunity debt leading to an early off-season RSV epidemic in 2021. A stronger dependence of infection susceptibility on immunity from previous exposure increases the size of the resurgent season. The early onset of the RSV resurgence in 2021, its marginally increased size relative to previous seasons and its decline by January 2022 suggest a stronger dependence of infection susceptibility on age-related factors, as well as a weaker effect of seasonality and a higher baseline transmissibility. The pattern of resurgence has been complicated by contact levels still not back to pre-pandemic levels. Further fitting of RSV resurgence in multiple countries incorporating data on contact patterns will be needed to further narrow down these parameters and to better predict the pathogen’s future trajectory, planning for a potential expansion of new immunisation products against RSV in the coming years.

## 1 Introduction

Respiratory syncytial virus (RSV) is a globally widespread (Obando-Pacheco et al., 2018; Nair et al., 2010) endemic virus causing respiratory infections, and is the most common pathogen in children diagnosed with acute lower respiratory infections (ALRI) (Shi et al., 2017). Most symptomatic and severe cases are in children younger than 5 years, with infants in the first year of life the most heavily affected. In temperate regions RSV is highly seasonal as RSV epidemics tend to occur in the winter (Obando-Pacheco et al., 2018; Bloom-Feshbach et al., 2013).

Through SARS-CoV-2 targeted mitigation measures, RSV epidemiology has been substantially disrupted with very few cases reported during the typical winter season in 2020–2021 (National flu and COVID-19 surveillance reports, 2021; Oh et al., 2021). As non-pharmaceutical interventions (NPI) have been gradually relaxed throughout 2021, countries across the world have reported substantial off-season RSV activity (Yeoh et al., 2020; Summeren et al., 2021; National flu and COVID-19 surveillance reports: 2021 to 2022 season, 2022; Tang et al., 2021; Opek et al., 2021). The disruption of RSV seasonality and the build-up of a large cohort of RSV-naive children poses great uncertainty about the public health burden the ongoing and upcoming RSV seasons will bring. Recent modelling studies analysed how the build-up of immunity debt due to NPIs could result in earlier and larger outbreaks of RSV (Zheng et al., 2021) and other respiratory pathogens (Baker et al., 2020) following the lifting of contact restrictions, and possibly affecting seasonality for some years to come.

The magnitude of the disruption in RSV epidemiology in future years will depend on transmission characteristics including the relative importance of respiratory tract development and general immune maturation with age versus the RSV-specific immunity built up by repeated exposure. The rate that immunity to reinfection wanes and the seasonal effects of more indoor contacts and changed climatic conditions in the winter also play a role (Li et al., 2022a). All of these factors are only partially understood. RSV disease burden rapidly declines in older children and it has been postulated that this is primarily due to immunity acquired by repeated exposure (Hodgson et al., 2020; Hogan et al., 2016; Yamin et al., 2016). This has been difficult to assess however, because the clockwork-like seasonality of RSV in most countries with routine surveillance meant that each year’s birth cohort was exposed to very similar infection risk making it impossible to disentangle the differential effects of development with age (mainly the development of the respiratory tract) and the RSV-specific immunity from past exposure. The suppression of RSV in the 2020–2021 season removed exposure while leaving ageing unchanged, decoupling these two factors and thereby creating an opportunity to determine their role in immunity acquisition. Immunity against reinfection with RSV has been estimated to last from 7 months (Arenas et al., 2009) to more than a year (Pan-Ngum et al., 2017), further complicated by partial heterologous immune evasion by its subtypes A and B (Human et al., 2020). Seasonal changes in climatic conditions can alter RSV’s effective transmissibility, modulated further by the concurrent change in interpersonal contact patterns shaping human to human transmission; however, the extent to which this pre-determines the timing of RSV seasons is largely unknown (Pitzer et al., 2015) with the off-season resurgence of RSV in Europe and elsewhere in 2021 (Li et al., 2022b; Eden et al., 2022) highlighting the potential for out of season spread.

Off-season outbreaks in 2021 were of different cumulative size and timing, although in almost all cases occurred weeks or months before the usual onset of the RSV season. While it’s clear that the build-up of susceptibility during a period of suppression due to NPIs played a role, it is less clear how the epidemiologic parameters of RSV would impact the peak and cumulative size of the resurgence. While this will also depend on the specifics of NPIs enacted in different countries and the recovery of contact patterns thereafter, the features of the resurgence also reflect incompletely understood aspects of RSV epidemiology, such as the role of age-dependent (respiratory tract and general immune system) development versus immunity from previous RSV infections in children. Modelling studies have made various assumptions on how susceptibility to RSV disease depends on these two factors (Zheng et al., 2021; Hodgson et al., 2020; Hogan et al., 2016, 2017; Pan-Ngum et al., 2017; Poletti et al., 2015; Kombe et al., 2019), as well as on the duration of post-infection immunity. We expect that depending on the true value of these parameters, the resurgence will take different forms, although it would inevitably be complicated by location-specific factors of contact rate recovery. In this study we use a mathematical model of RSV transmission calibrated to case and contact data from the United Kingdom to explore how these different aspects of RSV epidemiology shape post-suppression RSV dynamics and what we can conclude from the patterns of resurgence so far observed.

## 2 Methods

### 2.1 Model structure

**Fig. 1:**
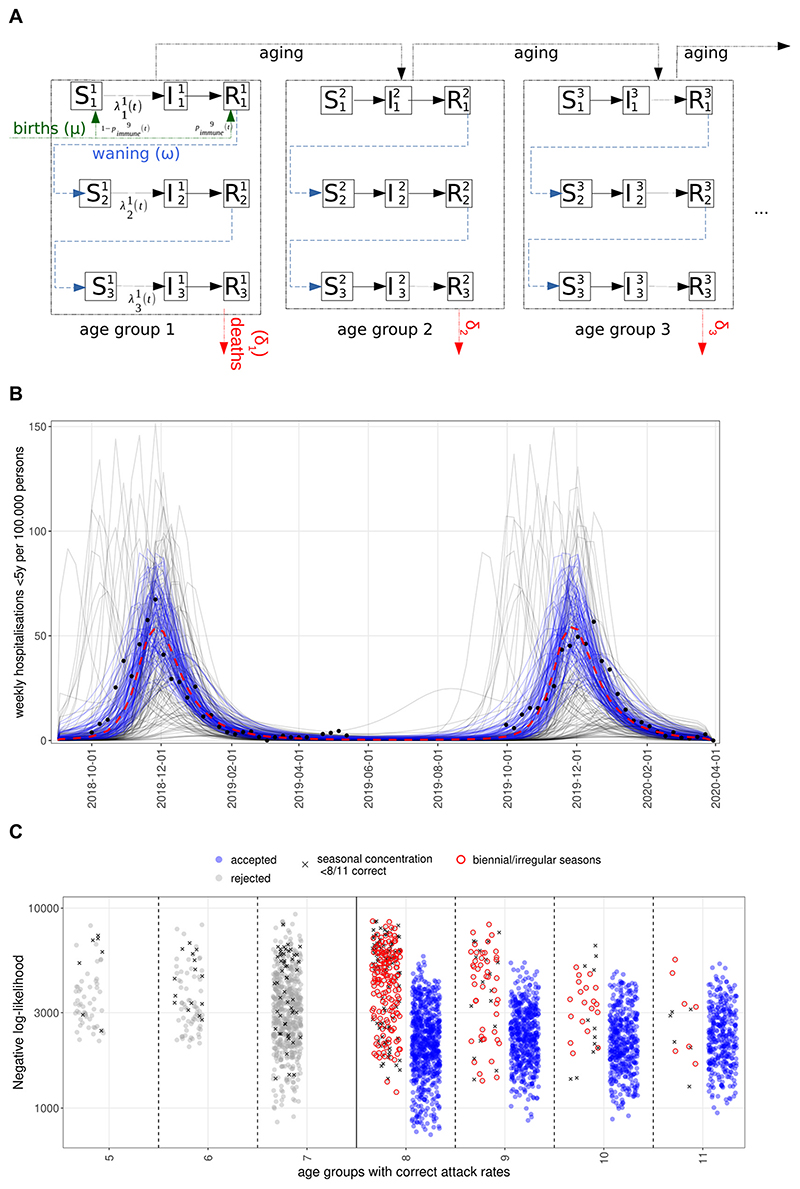
A. Age-structured SIRS model of RSV transmission with reinfections, immunity waning, births with maternal immunity and deaths. B. Simulated hospitalisations for children under 5 years before the COVID-19 pandemic. Blue lines are accepted simulations (see Methods) with a negative log-likelihood lower than 1500 (corresponding to the best 15 % of fits, red dashed lines showing their median); grey lines are those discarded. Black dots show hospitalisation rates for England from SARI Watch (National flu and COVID-19 surveillance reports: 2021 to 2022 season, 2022). C. Likelihoods of accepted and rejected parameterisations. The x-axis shows the number of age groups the simulations correctly predict the attack rate. Dots highlighted in red were rejected because of a biennial seasonal pattern and those marked with ‘x’ because of less than 85% of cases within the RSV season (week 40–13).

We developed an age-structured, deterministic compartmental SIRS-type dynamic transmission model (Fig. 1A) of repeated RSV infections (Hodgson et al., 2020). The model comprises 11 age groups (SI Table 1) with higher resolution in the first 2 years of life, where most severe disease is concentrated.

The model keeps track of up to three successive RSV infections, after which further reinfections still occur but are no longer distinguished, thus assuming that subsequent reinfections will not provide additional protection. Individuals are either susceptible (S), infected and infectious (I) or recovered (R) with short term sterilising immunity to reinfection. Following recovery from infection, individuals lose immunity at a rate ω to become susceptible again but with additional lifelong partial protection (reflected by a lower susceptibility to infection) gained from previous exposure. To reflect that mothers with a recent infection transfer antibodies to their children transplacentally (Chu et al., 2017) we assume that a proportion of newborns, determined by the proportion of adults of childbearing age with short term post infection immunity (ie. in the compartment R), will be born in the same immune state $\left({\text{R}}_{1}^{1}\right)$ as someone with a recent infection (Fig. 1 and SI Methods).

The model is initialised with a stationary population structure calculated from birth and death rates in England and Wales in 2020 (Deaths registered monthly in England and Wales - Office for National Statistics, 2022), so that the size of age groups is stationary already at the start of simulations (SI Methods). Simulations are then run for 30 years, which is sufficient (SI Fig. 6) to reach a stable pre-pandemic baseline seasonal pattern, before contact restrictions are introduced in 2020, followed by another 4 years of forward simulation following the lifting of NPIs in May 2021.

### 2.2 Contact rates (seasonality and NPIs)

To reproduce the seasonal dynamics of RSV transmission we modelled an annually recurring increase in the transmission rate (β) during the winter months (SI Methods). To model the suppression of RSV observed in the winter of 2020–21 we reduced transmission rates by 90% between 26/03/2020 and 17/05/2021, during which restrictive NPI measures were in place in the United Kingdom (COVID-19 Government Response Tracker, 2020), following which contact rates return to their pre-pandemic baseline. As a sensitivity analysis we also explored a scenario where contact rates start gradually recovering from 08/03/2021, linearly recovering to their baseline on 17/05/2021, instead of a step change on 17/05/2021.

### 2.3 Data sources for RSV incidence and fixed parameters

Our model parameters were informed by a combination of different data sources.

To match the overall seasonal pattern, we used case notifications from Respiratory DataMart (2013–2020) (Zhao et al., 2014), the Respiratory infections laboratory reports (2014–2020) (Respiratory infections: laboratory reports 2020, 2021) data series (SI Fig. 1), and rates of hospitalisations per population from SARI-Watch (2017–2022) (National flu and COVID-19 surveillance reports: 2021 to 2022 season, 2022). Hospitalisation counts for the < 5 y and 65 + y age groups from SARI-Watch were used to calculate the Poisson likelihood of simulated hospitalisations from week 40 of 2018 to week 20 of 2020 as age-stratified (Zhao et al., 2014) hospitalisation counts were only available for this (pre-pandemic) period.

Hospitalisation rates (per 100,000 population) were also available for the period following the emergence of COVID-19. While less severe than for case notifications, there is some level of under-ascertainment in the hospitalisation data, which we accounted for by taking the ratio of annual hospitalisations in SARI-Watch to estimates of the full RSV-associated annual hospitalisation burden in the literature (Taylor et al., 2016; Reeves et al., 2017, 2019; Fleming et al., 2015; Sharp et al., 2021).

The risk for hospitalisation upon infection was modelled specific to the age groups based on estimates from previous model fitting (Hodgson et al., 2020).

In addition, we used age-specific symptomatic attack rate estimates from a Kenyan household study (Munywoki et al., 2015).

Age-specific mixing was informed by a synthetic contact matrix for the United Kingdom (Prem et al., 2021).

Other fixed model parameters are the rate of recovery (γ) (Hodgson et al., 2020) from infection (set at 1/7 days^-1^) and the rate of births (2314/day) (Office for National Statistics, 2021).

### 2.4 Model selection

#### 2.4.1 Parameter sampling and selection by global criteria

As a non-notifiable disease, RSV surveillance data suffer from significant under-reporting and are subject to changes in testing rates and health seeking behaviour. Consequently, we decided to incorporate existing knowledge on age-specific attack rates in typical RSV seasons, the concentration of cases/hospitalisations within seasons (defined from week 40 to week 13) and the observed regularity of RSV in the UK (Fig. 1B, SI Fig. 1) for model selection.

Therefore, we filtered simulation results for validity by comparison with observed pre-pandemic RSV epidemiology, using three quantitative criteria that capture the essential characteristics of RSV epidemics in the UK:

1) The model has to reproduce the proportion of each age group infected in typical pre-pandemic RSV seasons. Accordingly, we retained those simulations only where the age-specific infection attack rates were within 0.5- to 2-fold of literature estimates (Munywoki et al., 2015), taking into account the large uncertainty in these–estimates, while noting that most simulations are closer to the median estimates (SI Fig. 3A).
2) more than 85 % of pre-pandemic infections have to occur within weeks 40 and 13 (inclusive), as in the RSV seasons from 2014 to 22020 in England and Wales (SI Figs. 1 and 3B, SI Table 5).
3) the relative difference between the dynamics of incident infections (SI Methods) of the last two pre-pandemic years had to be less than 15 % (SI Figure 7).

Simulations were retained when each of these criteria was satisfied in at least 8 of the model’s 11 age groups.

To analyse the effect of the parameters we conducted Latin Hypercube Sampling (LHS) with 20,000 sampling points (Table 1, SI Methods), within realistic ranges of the individual parameters and using probability distributions to ensure positivity where relevant.

We analysed the effect of five factors influencing RSV epidemiology on the expected post pandemic dynamics: (i) the relative effect of age and exposure on increasing immunity, (ii) the baseline (out of season) transmissibility (R_0_), the (iii) strength and (iv) duration of seasonally increased transmission, and (v) the waning rate of sterilising immunity post-infection.

**Fig. 2:**
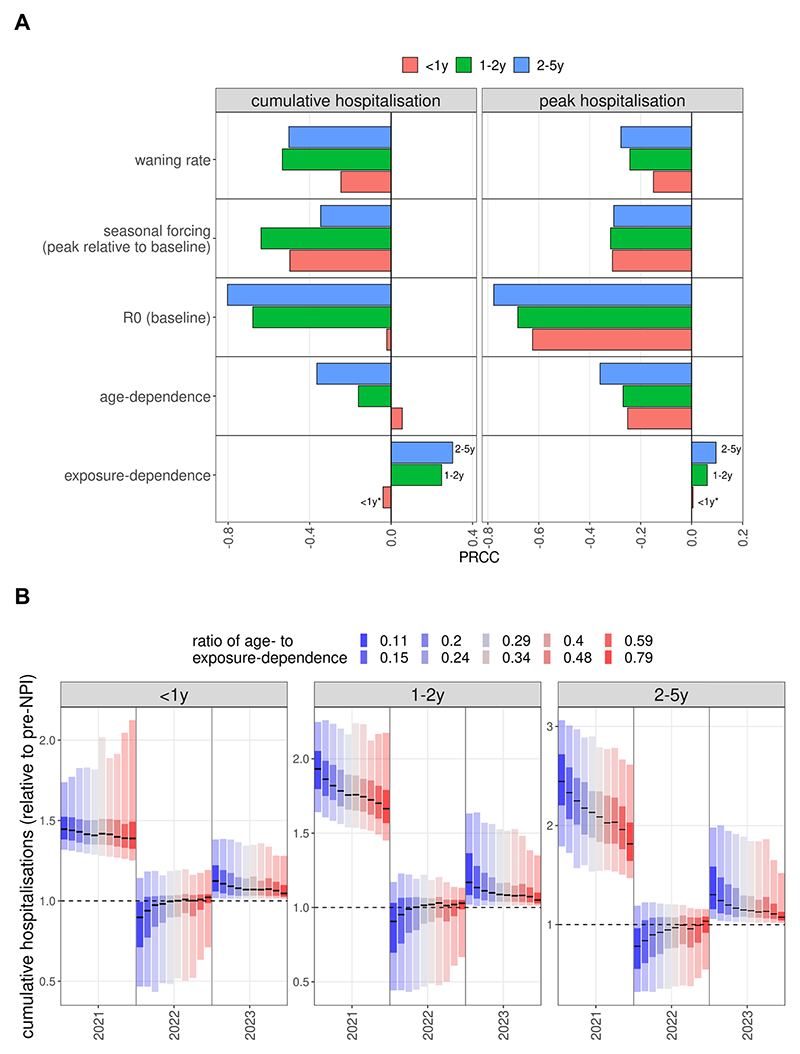
RSV resurgence as a function of epidemiological parameters. (A) Partial rank correlation coefficients (PRCC) between the sampled parameters and the proportionate change post-NPI in cumulative and peak hospitalisations. Asterisks show the correlation has a p-value above 0.05. (B) Level of cumulative hospitalisations as a function of susceptibility determined by previous exposure (blue) or age (red), captured by the ratio κ_age_/κ_exp_. Statistics calculated on the relative changes from pre-pandemic years to 2021 values (epi-years from week 23 to week 22).

The relative effect of age and exposure on immunity is described in the model by the susceptibility to infection decreasing exponentially as a function of both age and the level of exposure (Table 1, SI Fig. 2). The steepness of this decreasing function is defined by the two parameters *κ_exp_* and *κ_age_*, with larger values indicating a stronger dependence on exposure or age, respectively. After an initial sparser sampling of the parameter space, we found that all parameterisations showing seasonal dynamics consistent with UK data satisfy the condition *κ_exp_<* 1.65–4.5 *κ_age_, so parameter vectors outside this range were removed, increasing the proportion of realistic parameterisations to approximately 30 %. In the case of exposure-dependence, a value of *κ_exp_ =* 0.3 (lower bound of the explored parameter range, arbitrary units) means a 22 % reduction in the susceptibility after each infection whereas *κ_exp_* = 1.25 (higher bound) is equivalent to a 71 % reduction after each infection. In the case of age-dependence, *κ_age_=* 0.067 corresponds to a 7 % reduction in susceptibility by moving up one age group, meaning a 2-fold reduction from the youngest to the oldest age group. At the strongest age effect (*κ_age_ =* 1/3) the reduction is 28 % by each age group (28-fold from youngest to oldest). In *Results* we refer to these two parameters jointly by their ratio *κ_age_/κ_exp_*, with a higher value meaning stronger age dependence (Figs. 2B and 4).

#### 2.4.2 Likelihood calculations

In addition to the filtering of simulations by these global criteria, we also performed likelihood calculations to verify if results are comparable to a likelihood-based method. We compared simulated hospitalisations for the under-5 and over-65 year old age groups to the corresponding counts of hospitalisations in SARI-Watch for the 2018–2019 and 2019–2020 seasons (data provided by UKHSA, see Acknowledgements), taking into account under-ascertainment (SI Methods) and the variation in the size of catchment areas of reporting hospitals. We calculated a Poisson likelihood for each datapoint of this time series and summed the negative log-likelihoods.

We then added to this the sum of negative log-likelihoods calculated from comparing empirically observed age-specific attack rates (Munywoki et al., 2015) to the simulated ones, using binomial marginal probability (SI Methods).

#### 2.4.3 Sensitivity analysis by partial rank correlations

We grouped paediatric hospitalisations into three age groups: infants (below 1 year of age), children between 1 and 2 years of age, and children between 2 and 5 year of age. To explore how the relative (compared to the pre-pandemic seasons’ average, a value of 1 meaning the same burden as pre-pandemic) burden of the resurgent RSV season for these three groups correlate with the values of the sampled parameters we used partial rank correlation coefficients (PRCC) (Wu et al., 2013; Nunes et al., 2022), which quantifies the sign and strength of the parameters’ effect on model outputs. We used PRCC as a sensitivity measure because collinearities between parameters (SI Fig. 5) are controlled for when calculating the correlation coefficients. We used two measures of the disease burden, the cumulative number of hospitalisations and the peak level of hospitalisations. In both cases the calculations are by epi-years. For the burden calculations, we defined epi-years to last from week 23 of a given year to week 22 of the next year, so that off-season outbreaks in 2021 are within a single epi-year.

## 3 Results

Of the 20,666 simulations, 2621 were discarded because both the attack rate and seasonal concentration of cases were outside the desired range, an additional 9190 because of a mismatch in attack rates alone and 862 because of insufficient seasonal concentration of cases. A further 1895 simulations were discarded because of irregular annual epidemics. The remaining 6098 simulations reproduced the main features of pre-pandemic RSV epidemiology in the UK (Fig. 1B) in terms of age-stratified attack rates (SI Fig. 3A) and seasonal dynamics (SI Fig. 3B).

We then investigated if this filtering methodology is consistent with likelihood calculations. Accepted parameterisations have a more than 2-fold lower median negative log-likelihood than rejected ones (SI Fig. 4A-B), with the separation stronger for the likelihoods calculated from attack rates and hospitalisations for 65 + year olds. While there is overlap in the total likelihood between accepted and rejected simulations, their density functions are well separated (SI Fig. 4B). Rejected parameter sets that have a low negative log-likelihood suffer from incorrect attack rates in more than 3 age groups or show strongly biennial patterns (Fig. 1 C), which is not the case in the UK.

The likelihood figures are dominated by merely two available years of hospitalisation counts and while the negative log-likelihood might be low for some rejected parameterisations, these do not recapitulate the seasonal regularity and the age-specific attack rate distribution that are important features of RSV epidemics. Conversely however, 80 % of accepted parameterisations have a negative log-likelihood below 3000 and parameterisations that have likelihoods below 1000 are almost exclusively ones accepted by our global criteria. In other words, the filtering criteria above is broadly consistent with a likelihood approach as well, while it removes some parameterisations that fail to capture essential features of pre-pandemic RSV activity.

### 3.1 Dynamics in the first year following the easing of contact restrictions

Simulations of post-NPI RSV resurgence, assuming that NPIs are dropped and social mixing immediately returns to pre pandemic intensity, consistently show a substantial increase in RSV hospitalisations in the first epi-year (week 23 to week 22) after a relaxation of NPIs.

Sensitivity analysis by partial rank correlation coefficients showed that a stronger dependence of infection susceptibility on immunity from previous infections positively correlated with the relative size (1 = same size as pre-pandemic seasons) of the resurgent outbreak (Fig. 2A), although only for children above the age of 1 year. Meanwhile, a stronger age-dependence of infection susceptibility has a negative effect on the cumulative and peak size of the resurgent season, when controlled for correlations between model parameters.

Using the ratio *κ_age_/κ_exp_* (age-dependence divided by exposure-dependence parameter) to compare simulations, we found that the expected post-suppression increase in cumulative hospitalisations for under 1-year olds is largely consistent (30–40 %), independently of whether susceptibility is mainly dependent on age-related factors or immunity due to previous exposure (Fig. 2B). This is also reflected by the PRCC for this age group between *κ_exp_* and the cumulative burden having a p-value above 0.05 (Fig. 2A). In contrast, cumulative hospitalisations for 1–2 year olds depend strongly on the *κ_age_/κ_exp_* ratio, showing a higher correlation and with a median increase of approximately 60 % in the most age-dependent and 90 % in the most exposure-dependent case (Fig. 2B). For the 2–5 year olds group this effect is even more pronounced, with the median increase ranging from 75 % to 130 %. This is in some sense expected, since it is in these age groups that many first and second infections did not occur in the winter of 2020–2021 due to suppression, and if susceptibility is primarily exposure-dependent then the resurgence is more pronounced in the 2–5 year old age groups, as it is amplified by a higher proportion of first (or second) infections.

As a result of this differential increase of cases in the 2–5 year old groups, the average age of paediatric (under 5-year) hospitalisation is expected to increase (SI Figure 8B) by 2–3 months, again amplified by a stronger dependence of susceptibility on previous exposure. Peak hospitalisation demand showed a similar trend in that larger increases were found if susceptibility to infection is more exposure dependent (SI Figure 8A).

### 3.2 Dynamics in subsequent seasons

**Fig. 3:**
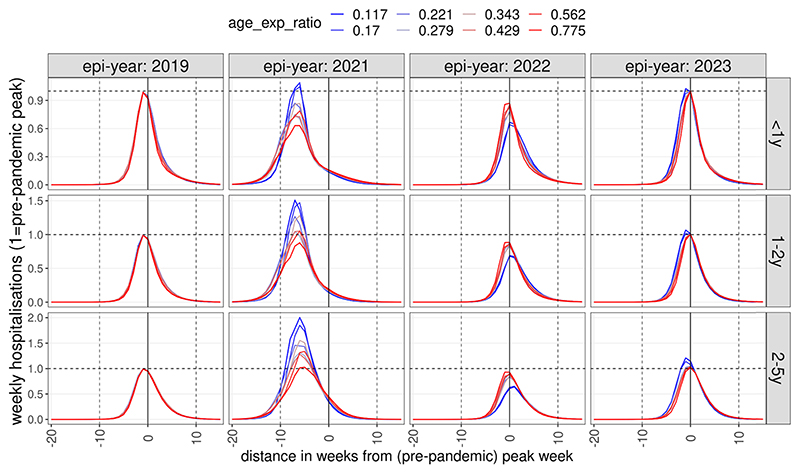
Dynamics of post-NPI weekly hospitalitalisation incidence as a function of infection susceptibility determined by age or previous exposure. Incidence was normalised to pre-pandemic peak incidence and time to the timing of the pre-pandemic peak. Colours indicate whether RSV immunity to infection is predominantly a function of previous exposure (blue) or age (red). The lines show median values by binned values of the ratio of the two parameters (κ_age_/κ_exp_).

Following the initial epidemic after contact behaviour returns to pre-pandemic levels, our accepted simulations suggest that in subsequent seasons the RSV burden would revert back to pre-pandemic incidence (Fig. 3A-B).

In scenarios assuming immunity to infection is largely dependent on age, RSV epidemiology in the 2022–2023 season was largely identical to that before the pandemic, if social mixing returned to pre-pandemic level following the removal of all NPIs in 2021. Assuming that infection risk is strongly dependent on previous exposure, however, led to a much larger epidemic in 2021 (1–2 y: 50 %, 2–5 y: 100 %, median values) and subsequently, in 2022, peak hospitalisation incidences were 30–40 % lower than pre-pandemic in children 1–2 and 2–5 years old (Fig. 3), respectively. In 2023 peak incidence marginally rebounded again above the pre-pandemic level for 1–2 and 2–5 year old children.

### 3.3 Comparison with observed resurgence in 2021

To explore the effect of parameters on resurgence in the general case, the scenarios above were based on the simplified assumption of contact levels returning to their pre-pandemic level stepwise in May 2021. This is likely not what occurred in reality (Jarvis et al., 2021), as contact rates started to recover from March 2021 when a phased reopening of schools started in England. Indeed, even the simulations with the best likelihoods could not reproduce well the resurgence of RSV observed from June 2021 (SI Figure 10) if making the unrealistic assumption of a stepwise recovery of contact rates.

**Fig. 4:**
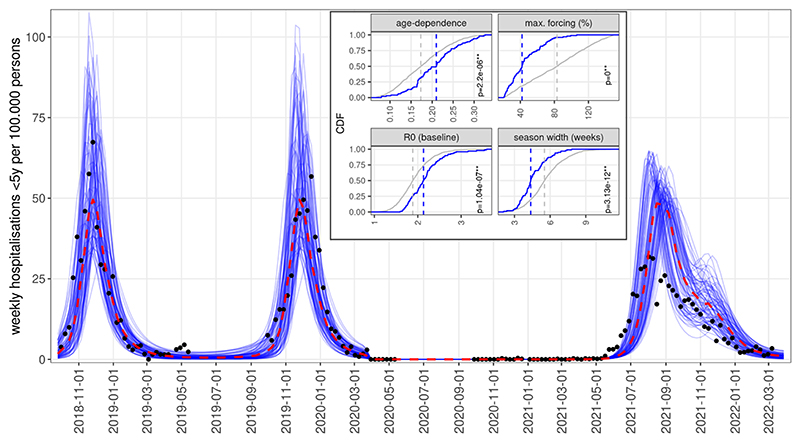
Simulations of RSV resurgence from June 2021, assuming gradual recovery of contact levels from March 2021, showing the 10% of simulations with the lowest error with respect to SARI-Watch hospitalisation rates. Inset: Cumulative density functions of parameters for all simulations accepted for matching pre-NPI RSV epidemiology (grey) versus the subset of accepted simulations that also replicated the early resurgence (blue). Dashed lines show median values for the two distributions; p-values are from Kolgomorov-Smirnov tests.

We therefore performed simulations with a gradual (linear) recovery of contact rates starting from the 8th of March 2021. As we did not have hospitalisation counts for this period, we calculated the Euclidean distance (mean squared deviation) of simulated hospitalisation rates (per 100,000 population) for children under 5 years from the reported SARI-Watch rates in 2021–2022. We found that the gradual recovery of contact rates from March resulted in several simulated trajectories showing a resurgence from June 2021, as was observed in reality (Fig. 4).

We further explored if these trajectories reproducing the early post-NPI resurgence significantly differed from all accepted simulations in their parameterisation. We found that four of the epidemiologic parameters indeed had statistically different distributions compared to all accepted parameterisations selected only on the basis of their pre-pandemic features (Fig. 4 inset). Specifically, the early resurgence scenarios showed a significantly stronger dependence of susceptibility on age-related factors, a significantly lower (median value 40%) seasonal forcing, a higher baseline transmissibility and also a shorter period of above-baseline forcing. These simulations also replicated the longer season duration, declining throughout the Autumn and ending in the early winter. The median value of the resurgent season’s peak was roughly equivalent to pre-pandemic seasons while a lower peak was observed in reality. This is likely due to the fact that we assumed a complete recovery of contact rates to the pre-pandemic level, while in reality contact levels did not recover yet in 2021 to their pre-COVID-19 level with continued high levels of homeworking and reduced use of public transport (Jarvis et al., 2021).

Despite the earlier resurgence in 2021, these simulations also largely reverted to their normal seasonality from 2022 (SI Figure 9).

## 4 Discussion

In 2021, RSV circulation in the UK and many other temperate settings started well ahead of the usual season, likely due to a build-up of a large pool of previously unexposed and therefore susceptible children. Using an age-structured dynamic model we explored how the pattern of RSV resurgence depends on its main epidemiologic parameters, first under the assumption that contact behaviour reverted to its pre-pandemic intensity in 2021. Within the range of plausible parameterisations we explored, we found that in the absence of contact restrictions, the 2021–2022 season would result in 51 % (median value across parameterisations) more hospitalised cases in all children under the age of 5 than in pre-pandemic years. This increase was particularly pronounced in children older than 1 year of age, leading to an increase in the average age of a hospitalised RSV case that season. We also found that uncertainty in the dependence of infection susceptibility on age or previous exposure had a large effect on the size of the resurgence, with a stronger dependence on previous infections leading to larger resurgent seasons, in particular in the 1–5 year old groups.

In reality, the cumulative size of the resurgent season in 2021 was only marginally larger than the seasons of 2018 and 2019, with 516 hospitalisations in 2021 versus 500 (2018) and 494 (2019) per 100,000 population (SARI-Watch) (National flu and COVID-19 surveillance reports: 2021 to 2022 season, 2022) and the peak size in 2021 smaller than of pre-pandemic seasons.

This is likely due to the fact that while the UK largely dropped any restrictions to contacts relevant to the transmission of RSV in Summer 2021, social contacts did not yet reach pre-pandemic intensity (CMMID, 2020) in 2021, with many continuing to work from home, reduced use of public transit and fewer larger indoor gatherings being organised.

By replacing our first assumption of contact recovering step-wise in May 2021 with a gradual recovery starting from March, we could reproduce the early onset of the RSV resurgence from June 2021. Analysing the parameter distributions of simulations matching the early resurgence and the epidemic’s decline in the autumn and early winter, we found that they are characterised by stronger age-dependence, weaker (40% above baseline) seasonal forcing and higher baseline transmissibility. Thus, the early resurgence and the absence of significant RSV activity in the winter of 2021–2022 suggest that the seasonal factors behind RSV’s regular annual pattern may be weaker than often assumed previously, while the baseline transmissibility of RSV is higher, and it only needs to be triggered by small changes in climate and contact patterns. However, this finding needs to be qualified by the fact that contact patterns did not fully recover and indeed continuously changed in 2021 and early 2022, with for instance mask wearing increasing in December 2021 during the Omicron wave (Jarvis et al., 2021).

RSV surveillance in many countries showed a common pattern of suppression of the 2020–2021 season followed by off-season outbreaks, including France (Casalegno et al., 2021) or the Netherlands (Teirlinck et al., 2021). In the southern hemisphere, Australia also saw a delayed (Yeoh et al., 2020; McNab et al., 2021) RSV epidemic in the summer months, starting 20 weeks later than usual and reaching a higher peak, confirmed both by surveillance testing and hospital admissions (Foley et al., 2021). As we recently showed in a Bayesian model fitting study (Krauer et al., 2022), the pre- and pandemic RSV data in the case of Australia (New South Wales) also support high baseline transmissibility and low seasonal forcing, similarly to our analysis here.

France, the Netherlands, Australia and Iceland all reported a substantial increase in the average age of infection for children, ranging from 2 to more than 10 months (Summeren et al., 2021). The delayed onset, the increase of RSV burden in the 1–5 years age groups, as well as the increase in the average age of infection in these countries are consistent with our modelling above. However, it is also clear the peak level and duration of these resurgent RSV outbreaks were also modulated by retained NPIs, as well as contact patterns not having reverted to pre-pandemic levels and possibly changes in testing rates. A more systematic analysis across multiple countries will be required once data for the entire season is available to arrive at conclusions on the relative role of the epidemiologic parameters analysed in this study.

Our knowledge of what determines susceptibility to RSV disease in children remains incomplete. A longitudinal study in 1986 (Glezen et al., 1986) followed newborns until 5 years of age and found similar infection rates in infants and 1–2 year olds and that the risk of reinfection was reduced in the presence of RSV specific antibodies. However, Scott et al. (2006). showed through molecular analyses of a longitudinal houshold study in coastal Kenya that sterilising immunity against reinfection with either the same strain or the same group often does not last until the next RSV season. A birth cohort study including 635 children in Kenya (Ohuma et al., 2012) demonstrated a 70 % reduction in infection risk following the first and 59 % following second infection for about six months. They also found that disease severity was primarily age-rather than exposure-related. Human challenge studies in adults (Hall et al., 1991; DeVincenzo et al., 2010) showed strong dependence of reinfection risk with the presence of F and G antibodies from previous infection but that even with high antibody levels the risk of reinfection if challenged was 25 %. In summary, there are only a few studies that have assessed the relative role of age and previous infection in modulating paediatric reinfection risk. Most find a limited role of age and some short-lived protection from previous infection. However, other factors that can be context-specific, for example changing contact patterns as young children become more mobile and start attending daycare (Qian et al., 2021), may mask some of the age effects observed.

All modelling studies for RSV have to make assumptions regarding the change in susceptibility to infection as children age to account for the strongly age-specific clinical profile of RSV. Several studies assume reduced susceptibility following infection, with a range of a perpetual 25–70 % reduction in the susceptibility to subsequent infections, which was either a prior based on estimates derived from the literature (Zheng et al., 2021; Pan-Ngum et al., 2017; Poletti et al., 2015) or arrived at by fitting an exposure-dependent model (Hodgson et al., 2020). In some cases, an age-dependent reduction in susceptibility was assumed instead of exposure-determined immunity (Hogan et al., 2016, 2017). Kombe et al. (2019). considered the effect of both factors and jointly inferred them through fitting to data from a detailed longitudinal household study in Kenya. They assumed a similar exponential form as in our present study while also analysing infections with heterologous strains. For this setting they estimated a small (<10 %) age-dependent reduction in susceptibility to infection in 1–4 year olds relative to infants, and a > 70 % reduction in older age groups. Previous infections were estimated to permanently halve susceptibility to reinfection. Similar evidence from other settings is needed to get a better overarching picture on how immunity against RSV is shaped by ageing and infection history.

In recent years many mathematical modelling studies for RSV transmission have been conducted (Zheng et al., 2021; Hodgson et al., 2020; Hogan et al., 2016, 2017; Pan-Ngum et al., 2017; Poletti et al., 2015; Kombe et al., 2019, 2021; Mezei et al., 2021), especially in the context of modelling prospective public health interventions, laying the groundwork for the potential introduction of maternal vaccines (Madhi et al., 2020) and lower cost long-lasting monoclonal antibodies (Griffin et al., 2020) in the coming years. A number of recent modelling studies have also analysed the potential patterns of resurgence of RSV and other respiratory pathogens following the easing of COVID-19-related restrictions (Zheng et al., 2021; Baker et al., 2020). Baker et al. (2020) raised the possibility of enlarged post-NPI outbreaks of RSV and influenza due to the increase of susceptibility caused by immunity loss during NPIs, predicting peak outbreaks in the winter of 2021–2022 and outbreak size positively correlated with the duration and stringency of restrictions. Zheng et al. (2021) used an age-structured SIS model to predict that the buildup of susceptibility during NPIs will lead to an earlier and larger RSV season in 2021–2022 and an increase in the average age of infection. Our findings are consistent with both studies, while we in addition also explored the underlying dependence on the key parameters of RSV epidemiology in such forecasts, in particular the role of age-related factors and immunity from previous exposure in children under 5 years.

The increasing availability of post-NPI RSV data from more and more countries in the coming months provides an opportunity to further study the relative importance of age and exposure in RSV transmission dynamics with statistical inference methods. Our study highlights the importance of a better understanding of such for predicting RSV epidemiology following the interruption of transmission due to COVID-19 restrictions. Similar considerations will apply following a likely partial interruption of RSV transmission as RSV vaccines currently undergoing clinical trials are considered for routine infant immunisation in the years to come (Griffin et al., 2020; GSK, 2021; RSV Vaccine and mAb Snapshot, 2021; Pfizer, 2021).

## Supplementary Material

Supplementary data associated with this article can be found in the online version at doi:10.1016/j.epidem.2022.100614.

SI

## Figures and Tables

**Table 1:** Fixed and sampled parameters (distributions used for Latin Hypercube sampling).

Parameter name (symbol)	Values	Description
Susceptibility of the *i*th age group to *j*th infection $\left(\delta_{i}^{\left(j\right)}\right)$	$\delta_{i}^{\left(j\right)}=\delta_{0}\text{exp}\left(-\left(\kappa_{\text{exp}}j+\kappa_{age}i\right)\right)$ *κ_exp_* = Unif (0.3, 1.25) *κ_age_* = Unif(1/16, 1/3)	*κ_exp_* defines how the susceptibility to infection depends on whether it is the 1st, 2nd or 3rd infection *κ_age_*: defines how the susceptibility to infection depends on age *δ*_0_: scaling constant, to ensure parameterisations have the same *R_0_*
Seasonal forcing width (weeks)	Gamma(shape=10,rate=2) (minimum: 1, maximum: 14, median: 4.8)	Standard deviation of normal distribution defining the seasonal forcing (see SI Methods)
Seasonal forcing strength (maximum above baseline)	Unif(0.2,1.5)	Maximum level of seasonal forcing above a baseline of 1
Baseline transmissibility (*R*_0_)	*R*_0_=Gamma(shape=14,rate=8) (Spencer et al., 2021) (min: 0.48, max: 4.14)	*R*_0_ value at the baseline level (1) of seasonal forcing
Waning rate (ω)	ω = 1/Norm(mean=350,sd=50) (Hodgson et al., 2020; White et al., 2005; Moore et al., 2014)	Rate of loss of immunity post-infection (1/day)
rate of recovery (γ)	1/7 day^-1^ (Hodgson et al., 2020)	Rate of recovery from infection
birth rate	2314 births/day (Deaths registered monthly in England and Wales - Office for National Statistics, 2022)	Number of live births per day

## Data Availability

All simulations and plotting were implemented in R (R: The R Project for Statistical Computing, 2021), scripts available at: https://github.com/mbkoltai/RSV-resurgence-model. Figures in the main text and the SI can be reproduced by the script reproduce_results.R at the repository. The required inputs are contained in the folder repo_data/.
