# Supplementary material for: Determinants of RSV epidemiology following suppression through pandemic contact restrictions
Source: Epidemics. Author manuscript; Available in PMC 2022 Aug 5. (PMC9301974; doi:10.1016/j.epidem.2022.100614)
Supplement: SI [file EMS151984-supplement-SI.pdf]

Supplementary Figures

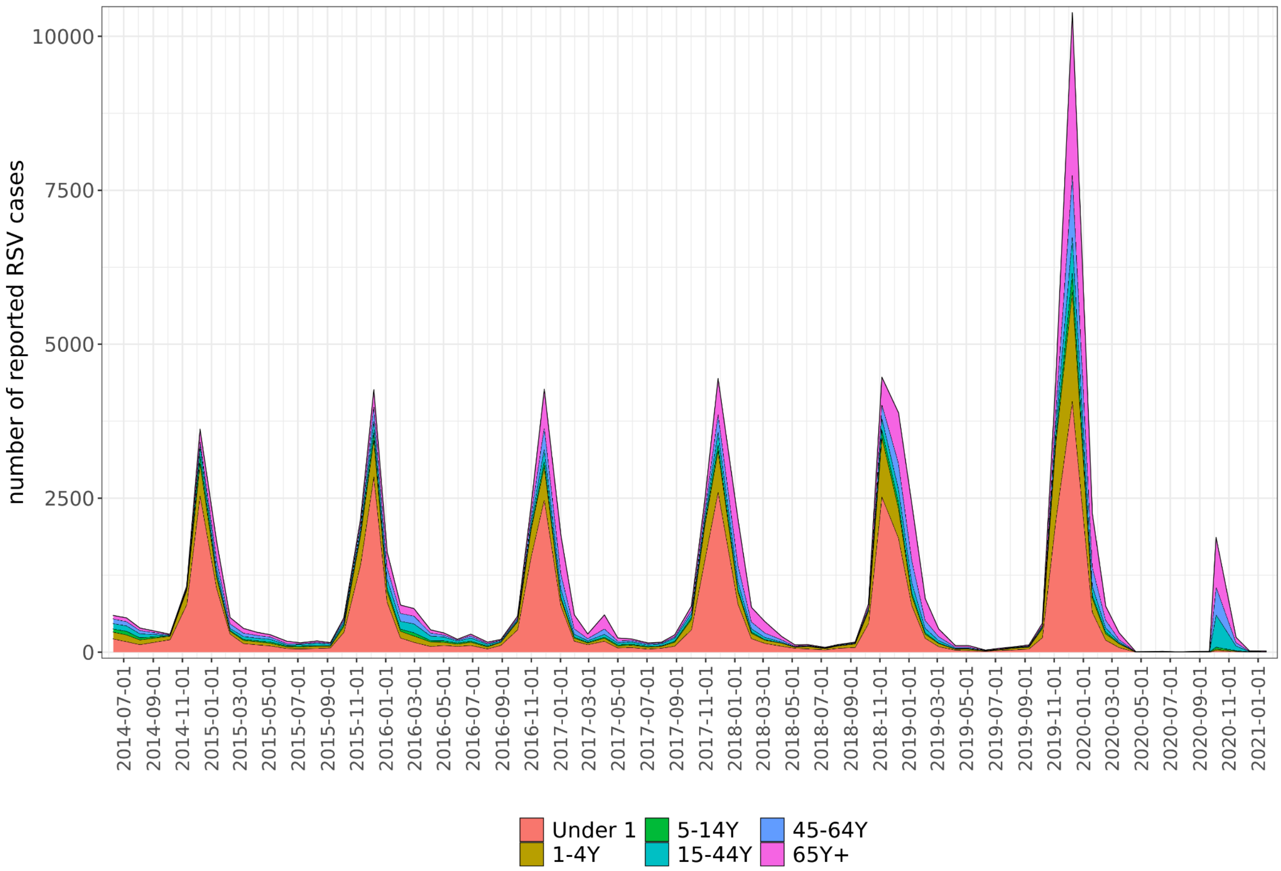

SI Figure 1 Reports of RSV infections from PHE and NHS laboratories in England and Wales, stacked by age groups, from 2014 to 2021, in 4-week reporting periods [65]

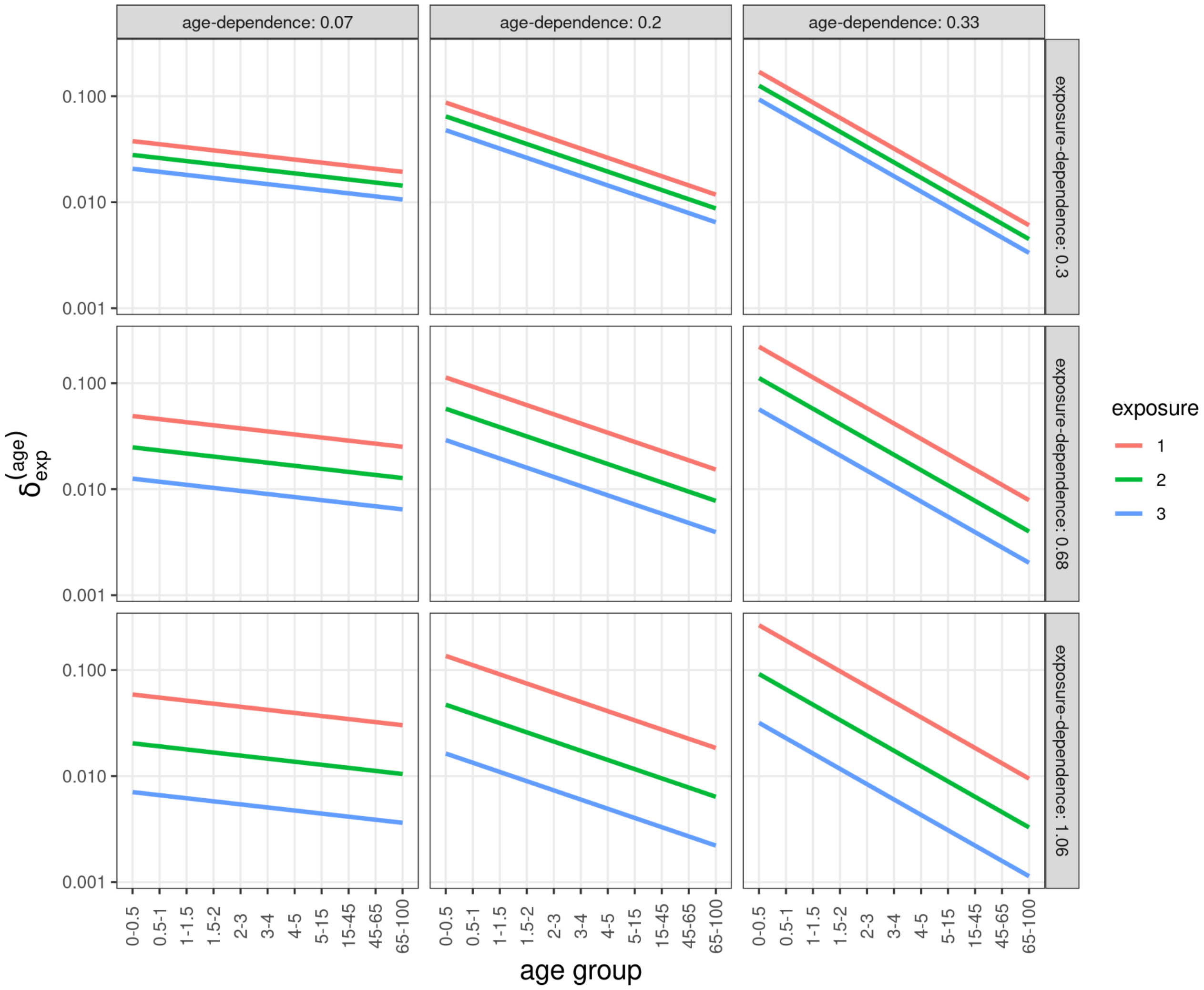

SI Figure 2 *Relative susceptibility ($\delta_{exp}^{(age)}$) to RSV infection as a function of exposure and age at different levels of dependence on exposure and age.*

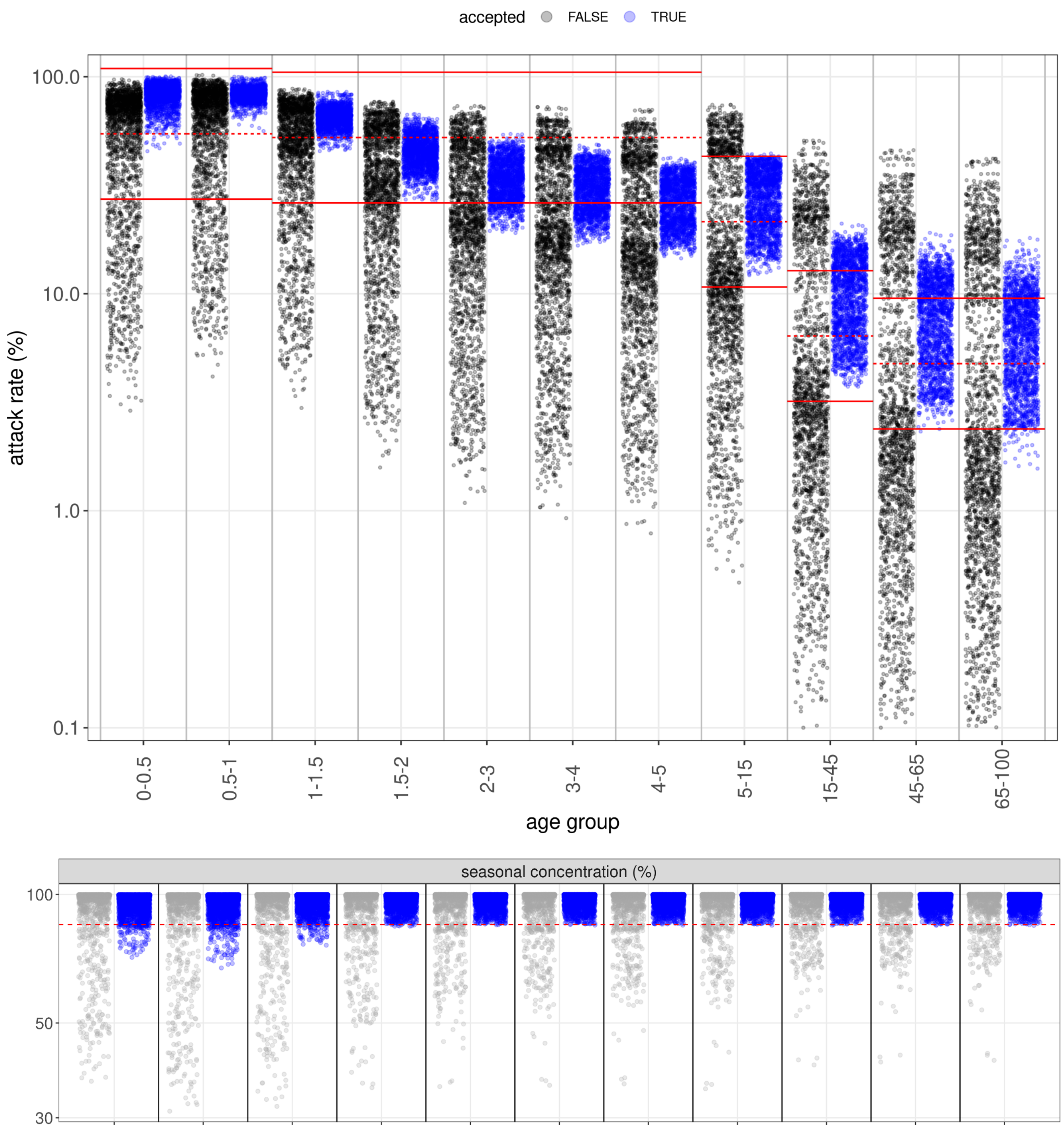

SI Figure 3 A Comparing literature estimates (red lines; dashed lines are median estimates, solid lines 0.5x and 2x of median) for pre-2020 annual cumulative infections to simulated values. Accepted simulations are in blue, rejected in black. For comparability with the attack rates in [37], we only counted first infections for the first age group. **B** Seasonal concentration of simulations with a negative log-likelihood less than 3000. Colour coding as in A.

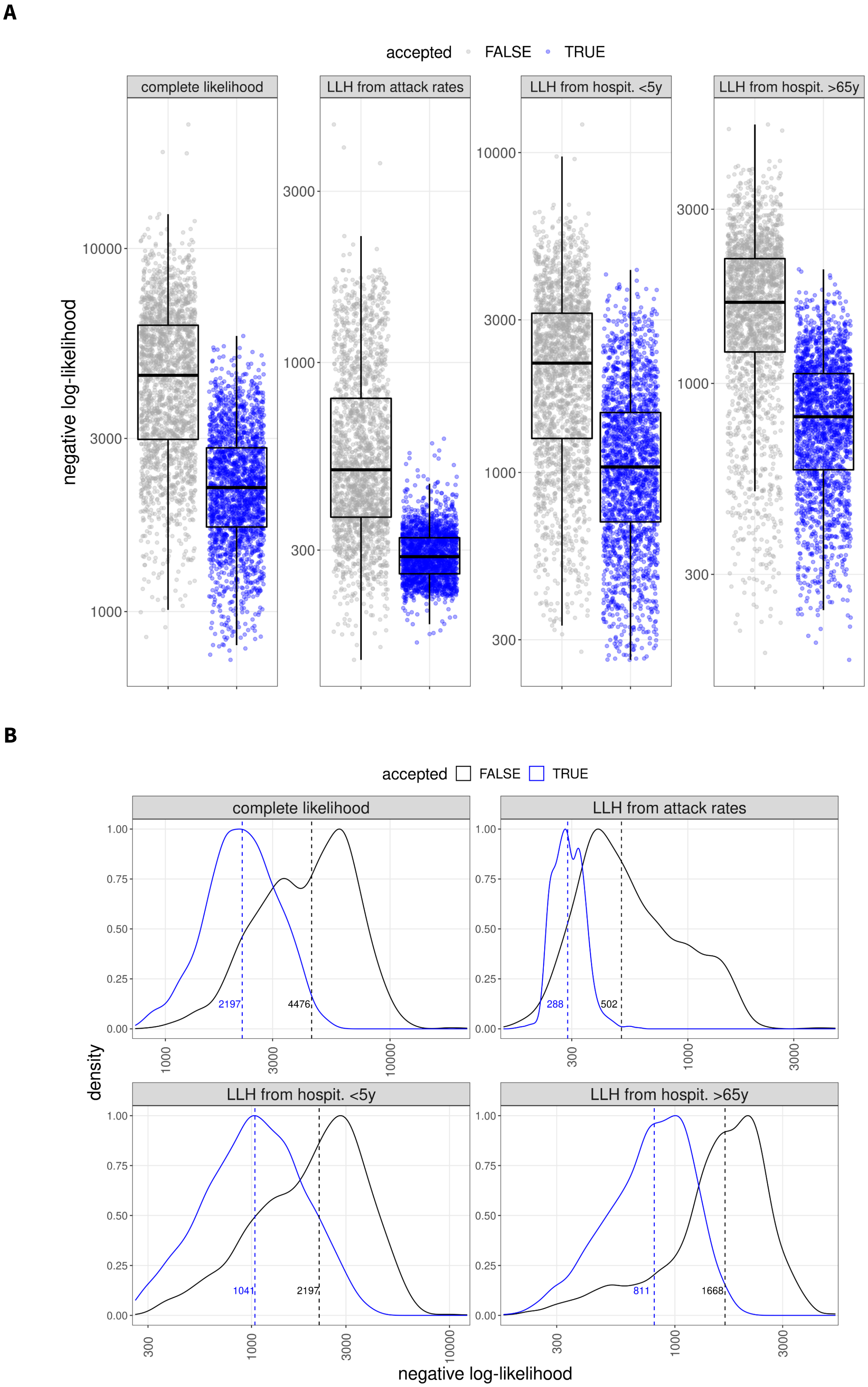
A
accepted FALSE TRUE
complete likelihood
LLH from attack rates
LLH from hospit. <5y
LLH from hospit. >65y
negative log-likelihood
10000
3000
1000
300
B
accepted FALSE TRUE
complete likelihood
LLH from attack rates
LLH from hospit. <5y
LLH from hospit. >65y
density
1.00
0.75
0.50
0.25
0.00
2197
4476
288
502
1041
2197
811
1668
300
1000
3000
10000
negative log-likelihood

C

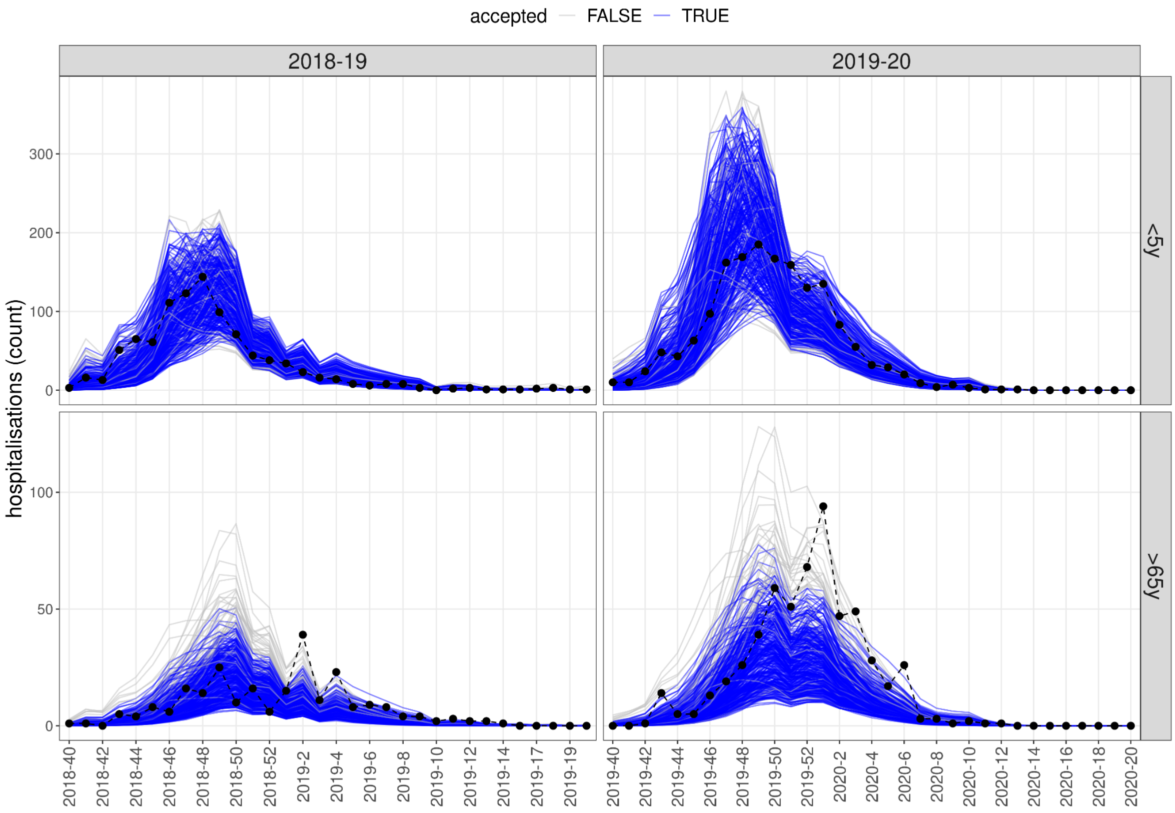

D

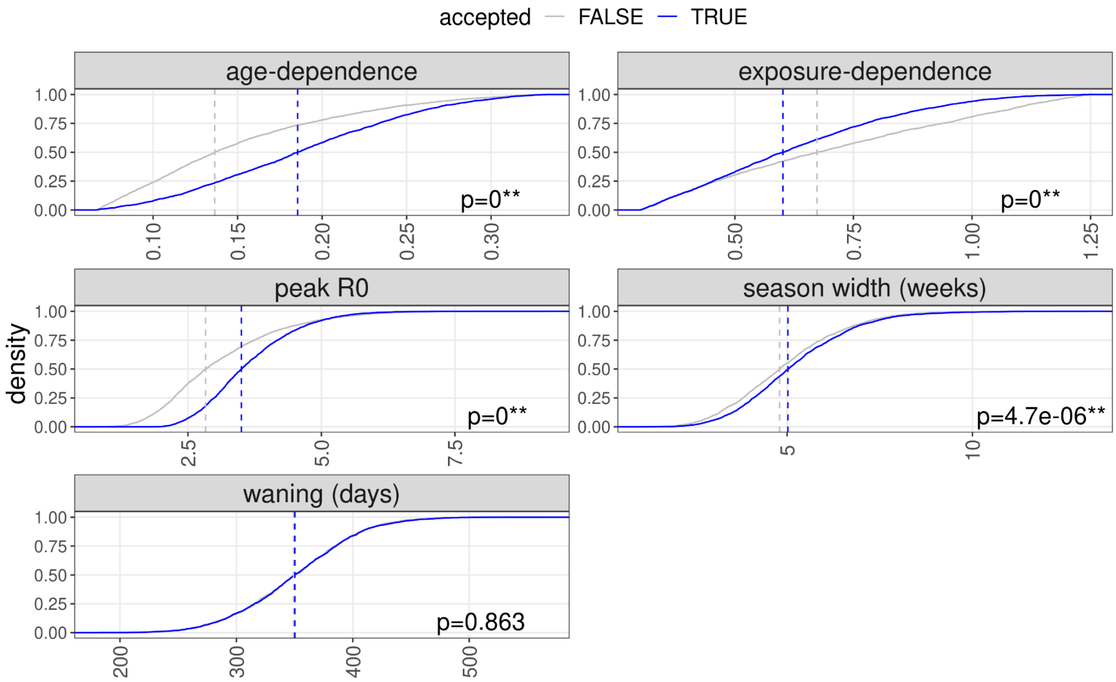

SI Figure 4: A Distribution of likelihood (negative log-likelihood) values for accepted and rejected simulations, with the likelihood's three components. **B** Density plot of likelihood values for accepted and rejected simulations. Dashed lines show medians. **C** Time series of hospitalisation counts and accepted/rejected simulations (likelihood below 1500). Counts vary with changing number/size of reporting hospitals, simulations scaled accordingly **D** Distribution

of parameter values for accepted and rejected simulations. P-values from Kolgomorov-Smirnov test.

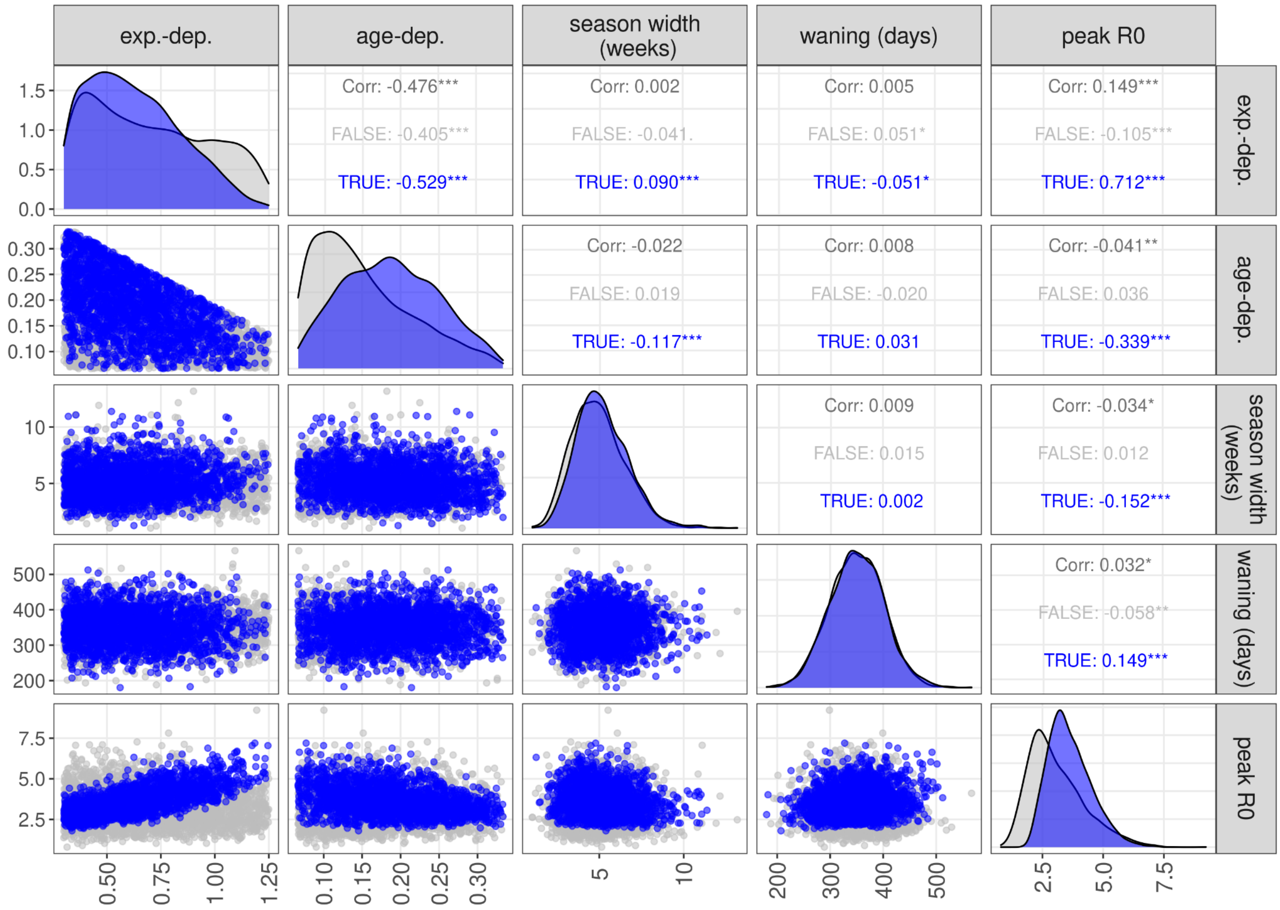

SI Figure 5. Correlations between parameter pairs. Blue dots are accepted, black dots rejected simulations.

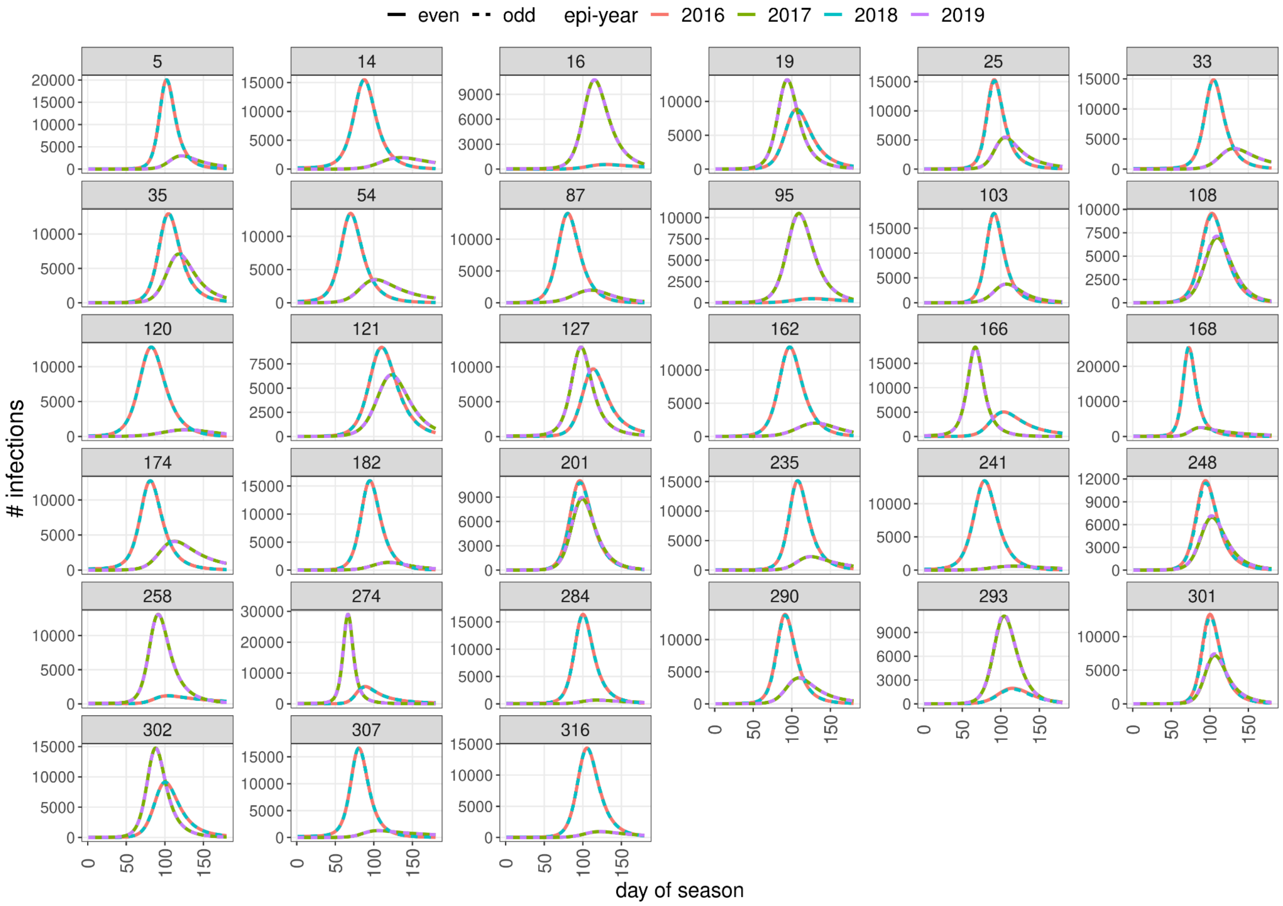

SI Figure 6 Incidence of cases in the 0 to 0.5 year age group for the years 2016-2019, for simulations showing a biennial pattern. Time is normalised to the onset of the season. Dashed and straight lines are odd and even years, respectively. Simulations have stabilised as there is no difference between the years 2016 and 2018 or 2017 and 2019.

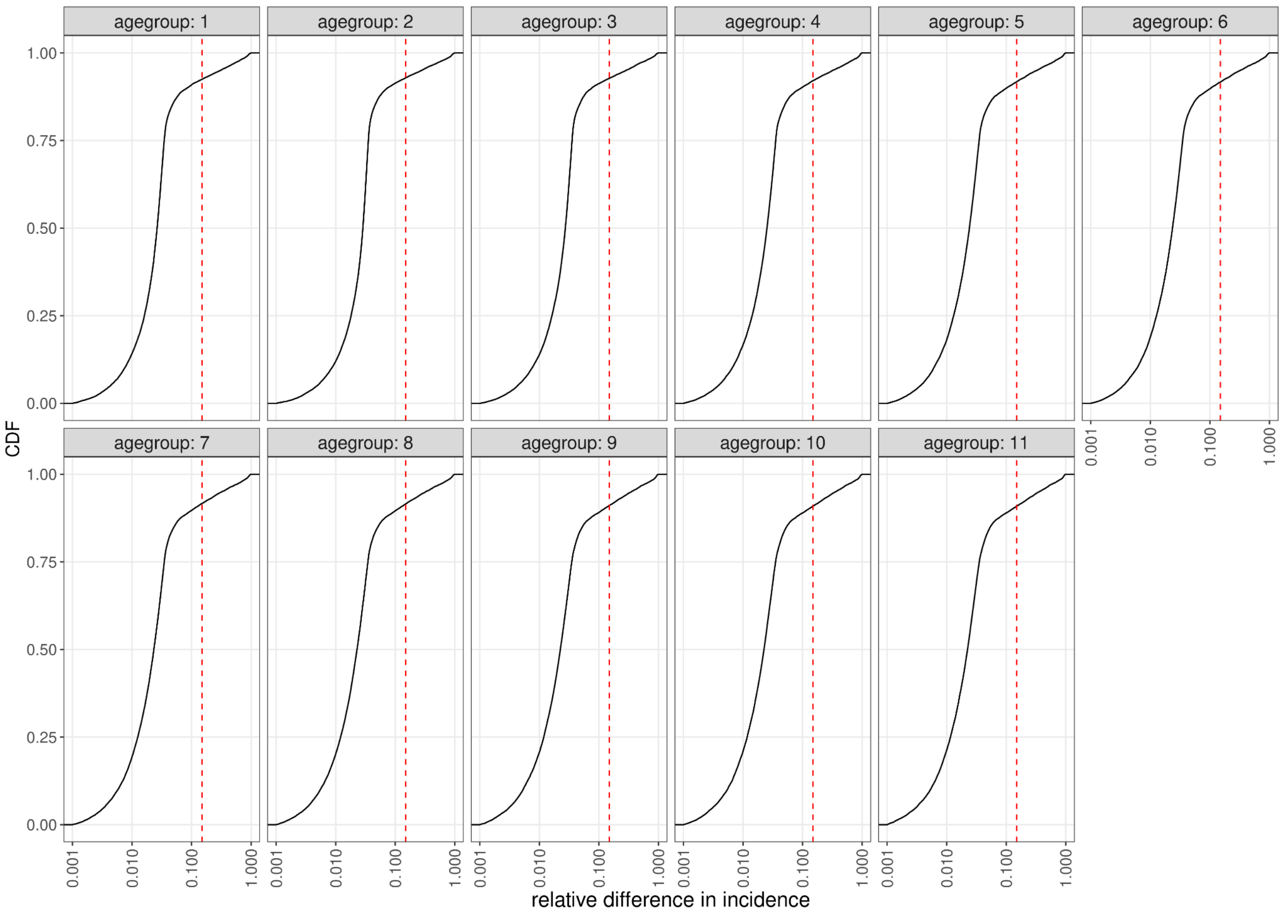

SI Figure 7 Cumulative density function of relative differences between 2018/19 and 2019/2020 RSV seasons, for the 7993 simulations selected by the first two criteria (SI Table 2). Relative difference is defined as the sum of (absolute values of) differences between matching days of the two seasons, normalised by the average cumulative incidence (SI Methods). Parameter sets to the right of the dashed red lines were removed, reducing the number of accepted parameterisations to 6098.

A

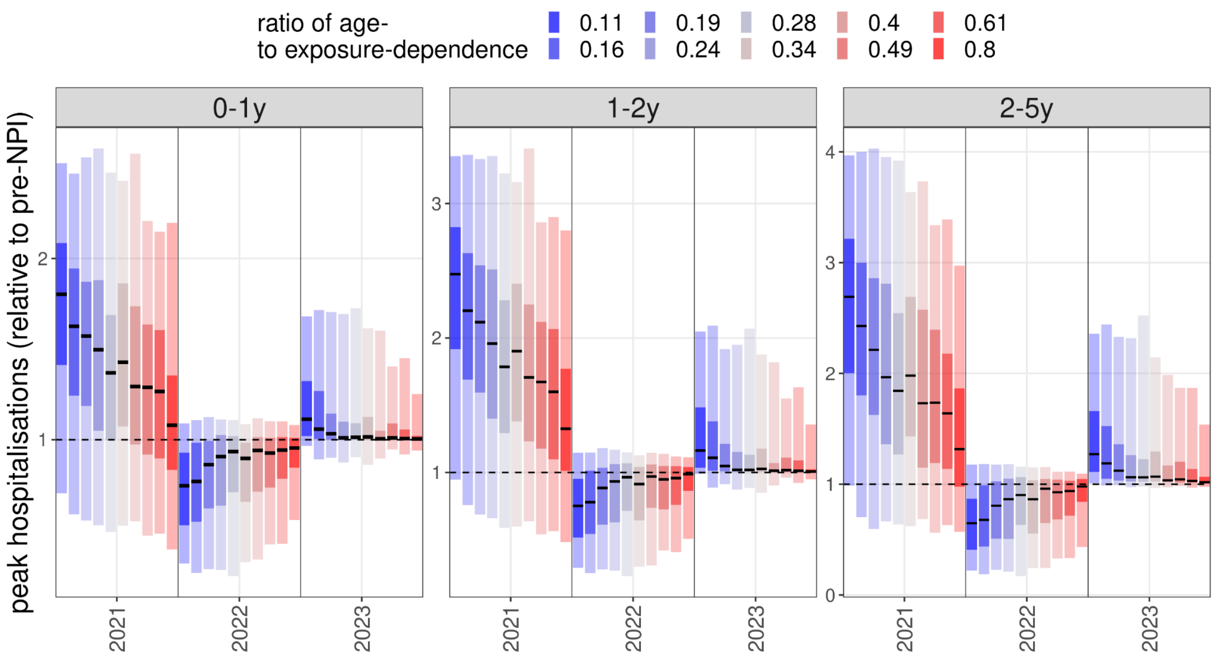

B

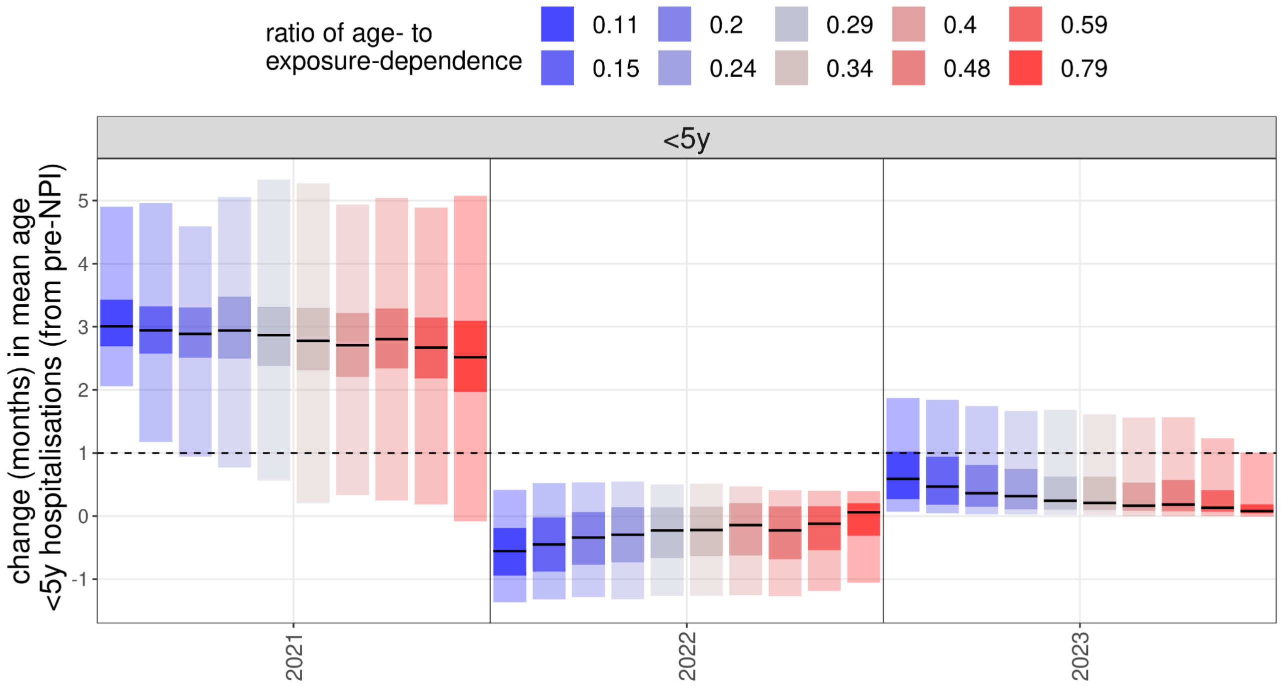

SI Figure 8: A Peak hospitalisations as a function of binned $\kappa_{age}/\kappa_{exp}$ values for accepted simulations, normalised to pre-pandemic peak values. **B** Change in the average age of under-5 hospitalisations as a function of binned $\kappa_{age}/\kappa_{exp}$ values for accepted simulations, normalised to pre-pandemic peak values.

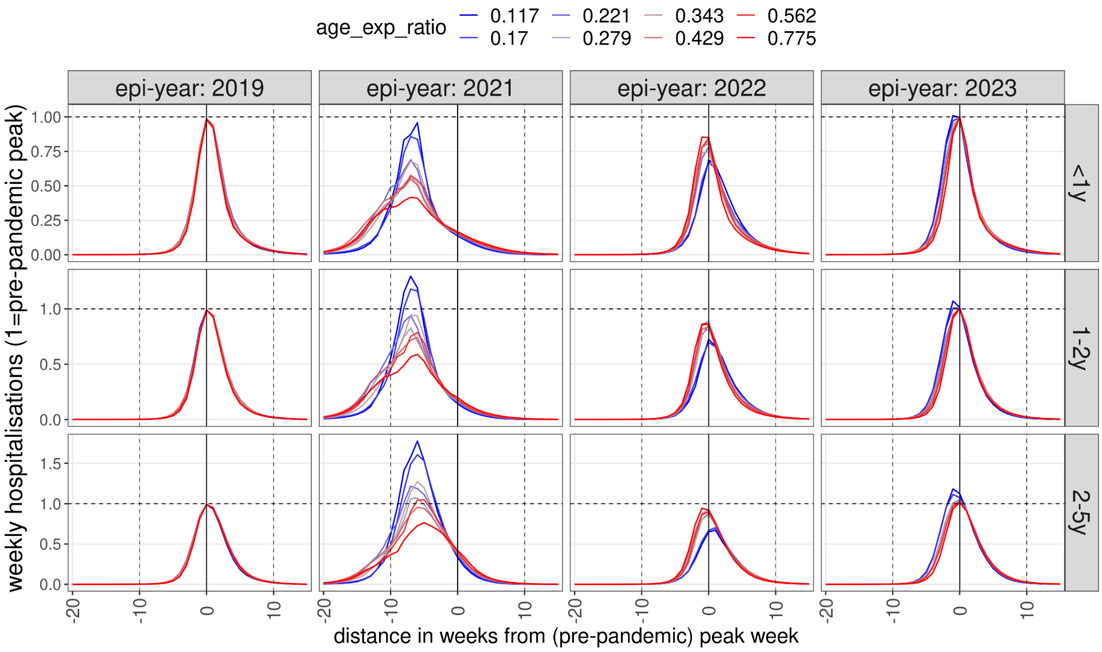

SI Figure 9 Dynamics of resurgence seasons as a function of binned $\kappa_{age}/\kappa_{exp}$ values for accepted simulations, assuming a gradual recovery of contact rates from March 2021 and showing the 10% of the simulations with the lowest error (mean squared deviation) with respect to SARI-Watch hospitalisation rates in 2022-22.

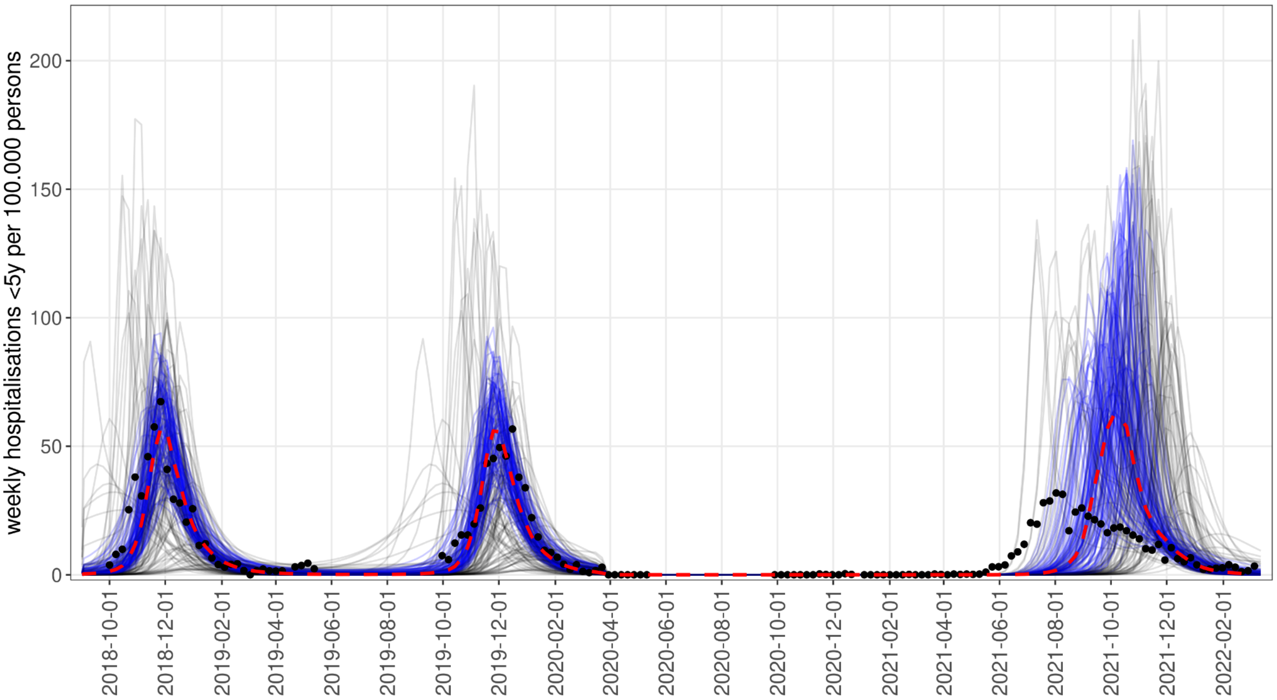

SI Figure 10 Simulations with the best likelihoods calculated from pre-pandemic data only (SARI-Watch count of hospitalisations 2018-19 and 2019-200, using a stepwise recovery of contact rates in May 2021

SI Methods

1. **Model structure and assumptions**
   - *1.1. Model structure*
   - *1.2. Model initialisation and external introductions*
   - *1.3. Susceptibility and disease severity*
2. **Processing simulation results**
   - *2.1. Parameter selection by regularity of annual seasons*
   - *2.2. Accounting for under-ascertainment of hospitalisation data*
   - *2.3. Likelihood calculations*

1. Model structure and assumptions

1.1 Model structure

We constructed an age-structured deterministic SIRS (susceptible - infectious - recovered - susceptible) model of RSV transmission. The model has 11 age groups with finer age strata in early childhood (Table 1 in main text) and with first, second and third infections as separate compartments. A compartment is defined by its type (S, I, R), age index and infection index: $S_i^j = S_1^2$ refers to susceptibles that have not been infected yet (i=1) and are in the second (2) age group. For susceptibles the infection index is the infection they *will* be exposed to, whereas for I and R it is the infection they are currently undergoing or have already recovered from, eg. R_1^2 refers to recovered individuals who have been infected once and are in the second age group (0.5-1 year olds).

Estimates of default parameter values and their source are shown in Table 1 of the main text. We used age demographics and the contact matrix [38] from the United Kingdom, as an example of a northern hemisphere country with strong seasonality of RSV (Figure 1B, SI Figure 1). The entries of the contact matrix were changed proportionately to the size of age groups relative to the original ones in [17], while also enforcing the contact matrix to be reciprocal.

Death rates from ONS data were slightly modified so that each age group has a stationary size close to the age structure in 2019 (SI Table 1). The proportions of the age groups as a percentage of the total population in the model and in ONS data have a deviation smaller than 0.3% for 9 out of 11 age groups and between 0.33% for the 45-65y group and 0.97% for the 65+ age group.

The model has 99 state variables in total, generated by the 3 types of compartment (S, I, R), 3 levels of infection and 11 age groups (3*3*11=99). We ordered variables by infection level, compartment type and age group, so the first 9 variables are $\left\{S_1^{(1)}, S_2^{(1)}, S_3^{(1)}, I_1^{(1)}, I_2^{(1)}, I_3^{(1)}, R_1^{(1)}, R_2^{(1)}, R_3^{(1)}\right\}$.

The system of 99 coupled ordinary differential equations (ODE) for the state variables consists of constant, linear and nonlinear terms, respectively.

The *constant* terms are births (μ), which is modelled as a constant inflow of new individuals into compartments S_1^1 and R_1^1.

To take into account maternal immunity we split the number of daily births between the compartments S_1^1 and R_1^1 as a function of the proportion of individuals in either the S (susceptible) or I (currently infected) compartments or in the R (recovered) compartments in the age group that is of childbearing age (15-45 years, 9th age group).

The proportion of births with maternal immunity, ie births into the compartment R_1^1 is

$$\mu\, p_{immune}(t) = \mu \frac{\sum_{i=1}^{n=3} R_i^9(t)}{\sum_{i=1}^{n=3} S_i^9(t) + I_i^9(t) + R_i^9(t)} \qquad \text{(SI Eq 1)}$$

and births without maternal immunity are $\mu\left(1 - p_{immune}(t)\right)$.

The *linear* terms in the ODEs are:

- waning, for R: $\omega\, R_i^j(t)$
- ageing, for any compartment X: $\frac{1}{365\, d^j} X_i^j(t)$, where d^j is the time spent in age group *j*
- deaths, for all compartments, in age group *j*: $\theta_j X_i^j(t)$
- recovery from infection, for I: $\gamma\, I_i^j(t)$

The *nonlinear* terms are the infection terms, a product of three terms: the force of infection (λ_i^j), the susceptibles getting infected, and the seasonal forcing, $\beta(t)$.

Dependence of the susceptibility to infection on age and exposure is a key control parameter in our modelling, therefore the force of infection is specific to the age group and/or the level of infection. For the *i*th infection of individuals in the *j*th age group, the force of infection is:

$$\lambda_i^j = \beta(t)\, \delta_i^{(j)} \sum_{k=1}^{n=11} \frac{\sum_{l=1}^{n=3} C_{j,k} I_l^{(k)}(t)}{N_k} \qquad \text{(SI Eq 2)}$$

Where $\delta_i^{(j)}$ is susceptibility to the *i*th infection for individuals in the *j*th age group.

N_k is the population of the *k*th age group and $\beta(t)$ is the seasonal forcing term. $C_{j,k}$ is the contact matrix from [38], rescaled proportionally for the age groups where the size (duration) of the age group was changed.

The entire infection term *F(t)* is then

$$F(t)_i^{(j)} = S_i^{(j)} \beta(t)\, \delta_i^{(j)} \sum_{k=1}^{n=11} \frac{\sum_{l=1}^{n=3} C_{j,k} I_l^{(k)}(t)}{N_k} \qquad \text{(SI Eq 3)}$$

The seasonal forcing term is, similarly to [15], a periodic equation with an annual peak similar to a normal distribution:

$$\beta(t) = \beta_0\left[1 + \rho\, exp\left(-0.5\left(\frac{abs(t-t_{peak})}{7\,\sigma}\right)^2\right)\right] \quad \text{(SI Eq 4)}$$

where σ is the season width in weeks (time *t* is in days) and ρ is the peak strength of seasonal forcing.

The full equations for the first age group are:

$$\dot{S}_1^{(1)}(t) = \mu\left(1 - p_{immune}(t)\right) - F(t)_1^{(1)} - \left(\frac{1}{365\, d^1} + \theta^{(1)}\right)S_1^{(1)}(t) \quad \text{(SI Eq 5.1)}$$

$$\dot{S}_2^{(1)}(t) = -F(t)_2^{(1)} - \left(\frac{1}{365\, d^1} + \theta^{(1)}\right)S_2^{(1)}(t) + \omega R_1^{(1)}(t)$$

$$\dot{S}_3^{(1)}(t) = -F(t)_3^{(1)} - \left(\frac{1}{365\, d^1} + \theta^{(1)}\right)S_3^{(1)}(t) + \omega\left(R_2^{(1)}(t) + R_3^{(1)}(t)\right)$$

$$\dot{I}_1^{1}(t) = F(t)_1^{(1)} - \left(\frac{1}{365\, d^1} + \theta^{(1)} + \gamma\right)I_1^{1}(t)$$

$$\dot{I}_2^{1}(t) = F(t)_2^{(1)} - \left(\frac{1}{365\, d^1} + \theta^{(1)} + \gamma\right)I_2^{(1)}(t)$$

$$\dot{I}(t)_3^{(1)} = F(t)_3^{(1)} - \left(\frac{1}{365\, d^1} + \theta^{(1)} + \gamma\right)I_3^{(1)}(t)$$

$$\dot{R}_1^{(1)}(t) = \mu\, p_{immune}(t) + \gamma I_1^{(1)}(t) - \left(\frac{1}{365\, d^1} + \theta^{(1)} + \omega\right)R_1^{(1)}(t)$$

$$\dot{R}_2^{(1)}(t) = \gamma I_2^{(1)}(t) - \left(\frac{1}{365\, d^1} + \theta^{(1)} + \omega\right)R_2^{(1)}(t)$$

$$\dot{R}_3^{(1)}(t) = \gamma I_2^{(1)}(t) - \left(\frac{1}{365\, d^1} + \theta^{(1)} + \omega\right)R_3^{(1)}(t) \quad \text{(SI Eq 5.9)}$$

For the age groups j>1, the equations have the same corresponding terms, except that there are no births, while there is an ageing term from the preceding age group. For example for $S_1^{(2)}(t)$:

$$\dot{S}(t)_1^{(2)} = -F(t)_1^{(2)} - \left(\frac{1}{365\, d^{(2)}} + \theta^{(2)}\right)S_1^{(2)}(t) + \frac{1}{365\, d^{(1)}}S_1^{(1)}(t) \quad \text{(SI Eq 6)}$$

To calculate incidence, we add 3x11 additional equations for new infections; for infection *i* and age group *j* the equation is:

$$\dot{C}(t)_i^{(j)} = F(t)_i^{(j)} \quad \text{(SI Eq 7)}$$

Incidence at time *t* is then calculated by the change in the value of $C(t)_i^{(j)}$ from *t-1* to *t*.

The equations are coded in R in vectorised format by the custom-made function *sirs_seasonal_forc_mat_immun()* contained in the file *essential_fcns.R*, in the subfolder *fcns/* at https://github.com/mbkoltai/RSV-model.

1.2 Model initialisation and external introductions

Simulations were started with the initial condition of the entire population in the susceptible compartments, except for 10 individuals per age group placed into the “first infection” compartment. We integrate the equations for 30 years before introducing the NPIs, and check whether the simulations have stabilised to an annual pattern.
Furthermore, to avoid RSV prevalence decaying to zero between seasons and to emulate the effect of external introductions, 10 new infections are introduced each month.

1.3 Susceptibility and disease severity

We use susceptibility to (re)infection as a composite parameter to capture how age and immunity affect the susceptibility to RSV disease. In reality, the observed decreasing clinical severity with age is likely to be a result of both lower susceptibility of infection reflected by lower attack rates [15,37] and infections being less severe due to higher immunity and a more developed immune and respiratory system. We used susceptibility to infection as a composite parameter to reflect these two effects to minimise model complexity.
Similarly, when calculating hospitalisation rates from the simulated number of cases we applied age-specific hospitalisation rates, but applied the same per-infection-probability to 1st, 2nd and 3rd infections within age groups. Using differential rates of severity would amplify the observed trends in the results, but would not fundamentally change them.

2. Processing simulation results

2.1 Parameter selection by regularity of annual seasons

As described in the main text, we discarded parameter sets that resulted in biennial or irregular patterns of RSV incidence pre-pandemic, only keeping parameter sets with stable (annual) oscillations. To do this, the relative difference between the two pre-NPI seasons were calculated as:

$$\int_{t=1}^{t=148} \left| incidence_{2018/19}(t) - incidence_{2019/20}(t) \right| /$$
$$mean([mean(incidence_{2018/19}), mean(incidence_{2019/20})]) \qquad \text{(SI Eq 7)}$$

The time index t here refers to the day *within* the RSV season, starting with week 40. The cumulative density function of this metric (by each age group) for the 7993 parameter sets is shown in SI Figure 7. We used a cut-off of 15% relative difference as an upper threshold for regular annual seasonality.

2.2 Accounting for under-ascertainment of hospitalisation data

There is likely some level of under-ascertainment in the SARI-Watch hospitalisation data that we used for likelihood calculations. To estimate this, we took the annual sum of hospitalisations in SARI-Watch and compared it to estimates of the full burden in the literature (SI Table 4). Specifically, we took the ratio of the annual burden from SARi_Watch and the mean of the annual disease burden estimates from the literature.

For the under-5 year old age group this resulted in an under-ascertainment rate of 52.1%, and for older adults (above 65 years) an under-ascertainment rate of 31%.
This calculation can be found in the *file fcns/load_SARI_data.R*.

2.3 Likelihood calculations

Likelihood values for hospitalisations were calculated by assuming that reported hospitalisation counts (from the SARI-Watch series, counts requested from UKHSA, see *Acknowledgements* in main text) at each timepoint *t* and age group *j* followed a Poisson distribution:

$hosp\_data_{j,t} \sim Poisson(k,\lambda)$ (SI Eq 8)

Since in this data series the number and size of hospitals reporting varies from week to week, we needed to scale the simulations ($hosp\_simul_{j,t}$) with the catchment area of the reporting hospitals at time point *t*. Since simulations were for the entire UK population, this scaling factor (r_{pop}) needs to be the ratio of the full population size of the relevant age group (under-5 and over-65 year olds) used in the model (4.18 million and 12.68 million, respectively).
Additionally, there is likely under-reporting in the hospitalisation data which we approximate by taking the ratio of the annual hospitalisation rates from SARI-Watch to the estimated full burden. Simulated hospitalisations were also scaled by this ratio (r_{und_rep}).
Then, to calculate the Poisson likelihood we have:

$p\_hosp_{j,t} = Poisson(k=hosp\_data_{j,t}, \lambda=r_{pop}*r_{und\_rep}*hosp\_simul_{j,t})$ (SI Eq 9)

The joint likelihood for all timepoints is then:

$$Pr(hosp\_data|hosp\_simul) = \prod_{t=1}^{65}\prod_{j=1}^{2} p\_hosp_{t,j} \quad \text{(SI Eq 10)}$$

In case of the time notation *t=1* means 01/10/2018 and *t=65* 11/05/2020, with weekly timesteps, whereas for age groups j={1,2} corresponds to under-5-year and over-65-year age groups.
Similarly, for attack rates, we assumed that the observed number of infections (n_{inf}) out of the sample size (n_{sample}) per age group follows a binomial distribution.
Then, the probability for the attack rate in age group *j*:

$p\_AR_j=Binom(k=n\_inf_j, n=n\_sample_j, p=AR\_simul_j)$ (SI Eq 11)

and the joint probability is

$$Pr(AR\_data|AR\_simul) = \prod_{j=1}^{11} p\_AR_j \quad \text{(SI Eq 12)}$$

We combine these probabilities by taking their negative log-likelihood, so the total likelihood is:

$$NegLL = -\left(\sum_{t=1}^{65}\sum_{j=1}^{2} log(p\_hosp_{t,j}) + \sum_{j=1}^{11} log(p\_AR_j)\right) \quad \text{(SI Eq 13)}$$

Because of the negative sign, a lower value means a higher likelihood.
